# Genetic Diversity and Evolutionary Analyses Reveal the Powdery Mildew Resistance Gene *Pm21* Undergoing Diversifying Selection

**DOI:** 10.3389/fgene.2020.00489

**Published:** 2020-05-12

**Authors:** Huagang He, Jian Ji, Hongjie Li, Juan Tong, Yongqiang Feng, Xiaolu Wang, Ran Han, Tongde Bie, Cheng Liu, Shanying Zhu

**Affiliations:** ^1^School of Food and Biological Engineering, Jiangsu University, Zhenjiang, China; ^2^National Engineering Laboratory for Crop Molecular Breeding, Institute of Crop Sciences, Chinese Academy of Agricultural Sciences, Beijing, China; ^3^Crop Research Institution, Shandong Academy of Agricultural Sciences, Jinan, China; ^4^Yangzhou Academy of Agricultural Sciences, Yangzhou, China; ^5^School of Environment, Jiangsu University, Zhenjiang, China

**Keywords:** *Dasypyrum villosum*, *Pm21* allele, genetic diversity, evolutionary analysis, diversifying selection, wheat powdery mildew resistance

## Abstract

Wheat powdery mildew caused by *Blumeria graminis* f. sp. *tritici* (*Bgt*) is a devastating disease that threatens wheat production and yield worldwide. The powdery mildew resistance gene *Pm21*, originating from wheat wild relative *Dasypyrum villosum*, encodes a coiled-coil, nucleotide-binding site, leucine-rich repeat (CC-NBS-LRR) protein and confers broad-spectrum resistance to wheat powdery mildew. In the present study, we isolated 73 *Pm21* alleles from different powdery mildew-resistant *D. villosum* accessions, among which, 38 alleles were non-redundant. Sequence analysis identified seven minor insertion-deletion (InDel) polymorphisms and 400 single nucleotide polymorphisms (SNPs) among the 38 non-redundant *Pm21* alleles. The nucleotide diversity of the LRR domain was significantly higher than those of the CC and NB-ARC domains. Further evolutionary analysis indicated that the solvent-exposed LRR residues of *Pm21* alleles had undergone diversifying selection (dN/dS = 3.19734). In addition, eight LRR motifs and four amino acid sites in the LRR domain were also experienced positive selection, indicating that these motifs and sites play critical roles in resistance specificity. The phylogenetic tree showed that 38 *Pm21* alleles were divided into seven classes. Classes A (including original *Pm21*), B and C were the major classes, including 26 alleles (68.4%). We also identified three non-functional *Pm21* alleles from four susceptible homozygous *D. villosum* lines (DvSus-1 to DvSus-4) and two susceptible wheat-*D. villosum* chromosome addition lines (DA6V#1 and DA6V#3). The genetic variations of non-functional *Pm21* alleles involved point mutation, deletion and insertion, respectively. The results also showed that the non-functional *Pm21* alleles in the two chromosome addition lines both came from the susceptible donors of *D. villosum*. This study gives a new insight into the evolutionary characteristics of *Pm21* alleles and discusses how to sustainably utilize *Pm21* in wheat production. This study also reveals the sequence variants and origins of non-functional *Pm21* alleles in *D. villosum* populations.

## Introduction

*Dasypyrum villosum* L. Candargy (2*n* = 2*x* = 14, VV), a diploid species native to the Mediterranean region, is an important wild resource for the improvement of common wheat (*Triticum aestivum* L., 2*n* = 6*x* = 42, AABBDD). *D. villosum* possesses good resistance to multiple wheat diseases, such as wheat spindle streak mosaic disease, eyespot, take-all, stem rust, stripe rust, and powdery mildew (Li and Zhu, 1999; De Pace et al., 2011; Wang et al., 2017). Four powdery mildew resistance (*Pm*) genes, *Pm21* (Chen et al., 1995), *PmV* (Li et al., 2005), *Pm55* (Zhang et al., 2016), and *Pm62* (Zhang et al., 2018), have been found in *D. villosum*. Among them, both *Pm21* and *PmV* are located on the short arm of chromosome 6V (6VS) and confer immunity to powdery mildew at the whole growth stages of wheat. *Pm55* and *Pm62* are mapped to the short arm of chromosome 5V (5VS) and the long arm of chromosome 2V (2VL), respectively, which provide powdery mildew resistance at the adult-plant stage.

*Pm21* was originally transferred from an accession of *D. villosum*, collected from Cambridge Botanical Garden, United Kingdom, to durum wheat (*T. turgidum* var. *durum* L.), and then a translocation line of wheat-*D. villosum* T6AL·6VS carrying *Pm21* was further developed (Chen et al., 1995). Using this translocation line as the powdery mildew resistance source, more than 20 varieties have been developed and released in the middle and lower reaches of the Yangtze River Valley and the southwest wheat-producing area, the most rampant areas of powdery mildew in China, where some *Pm* genes, such as *Pm2a* and *Pm4a*, are gradually losing their resistance (Bie et al., 2015a).

Undoubtedly, *Pm21* is a very valuable gene that confers highly effective resistance to tested isolates of *Blumeria graminis* f. sp. *tritici* (*Bgt*). However, no recombination occurs between the alien chromosome arm 6VS carrying *Pm21* and the wheat homoeologous chromosome arms, which limits the genetic mapping and the cloning of *Pm21* in the wheat backgrounds (Zhu et al., 2018). Recently, four seedling-susceptible *D. villosum* lines were identified from the natural populations. Based on the fine genetic map constructed, the gene *Pm21* was cloned and confirmed to encode a single coiled-coil, nucleotide-binding site, leucine-rich repeat (CC-NBS-LRR) protein (He et al., 2017, 2018).

In the present study, we isolated the *Pm21* alleles from different resistant *D. villosum* accessions and determined their genetic diversity, non-synonymous and synonymous substitution rates and positive selection sites. On the other hand, *D. villosum* germplasms susceptible to powdery mildew are rare, and only four susceptible *D. villosum* lines (DvSus-1 to DvSus-4) and two wheat-*D. villosum* chromosome 6V disomic addition lines (DA6V#1 and DA6V#3) were identified (Qi et al., 1998; Liu et al., 2011; He et al., 2017). Understanding the reason that these *D. villosum* germplasms keep or lose their resistance to powdery mildew will be useful to extend the effective duration of *Pm21* in agriculture. We also detected the sequence variations of *Pm21* alleles in the above germplasms for tracing their origins in natural population of *D. villosum*.

## Materials and Methods

### Plant Materials

*Dasypyrum villosum* accessions were gifted from Germplasm Resources Information Network (GRIN), GRIN Czech, Genebank Information System of the IPK Gatersleben (GBIS-IPK), and Nordic Genetic Resource Center (NordGen). The wheat-*D. villosum* chromosome 6V disomic addition lines DA6V#1 and DA6V#3 were provided by GRIN and Dr. Bernd Friebe (Kansas State University, Manhattan, KS, USA), respectively (Table S1). The *D. villosum* line DvRes-1 carries the original *Pm21* gene. DvRes-2 and DvRes-3 were derived from the powdery mildew resistant individuals of the accessions GRA961 and GRA1114, respectively. Lines DvSus-1 to DvSus-4 were derived from the susceptible individuals of the accessions GRA2738, GRA962, GRA1105, and PI 598390, respectively. The wheat variety (cv.) Yangmai 18 was a wheat-*D. villosum* translocation line that carries *Pm21*. The wheat cv. Yangmai 9 was susceptible to powdery mildew. Both of them were developed in Yangzhou Academy of Agricultural Sciences, Yangzhou, China. Plants were grown under a daily cycle of 16 h of light and 8 h of darkness at 24°C in a greenhouse.

### Evaluation of Powdery Mildew Resistance

*Blumeria graminis* f. sp. *tritici* (*Bgt*) isolate YZ01 is a virulent isolate collected from Yangzhou region (Jiangsu Province, China). All plants, *D. villosum* accessions or lines and wheat varieties, were inoculated with *Bgt* isolate YZ01 at one-leaf stage (He et al., 2016). The powdery mildew responses of plants were evaluated at 8 d after inoculation.

### Allelic Test

The susceptible homozygous *D. villosum* line DvSus-1 was used as female parent to cross with other susceptible lines, DvSus-2, DvSus-3, and DvSus-4, to produce three F_1_ hybrids, DvSus-1/DvSus-2, DvSus-1/DvSus-3, and DvSus-1/DvSus-4, respectively. The wheat-*D. villosum* chromosome 6V disomic addition line DA6V#1 susceptible to powdery mildew was crossed with another susceptible chromosome 6V addition line DA6V#3 to result in F_1_ hybrid DA6V#1/DA6V#3. All F_1_ plants derived from different crosses were inoculated with *Bgt* isolate YZ01 at one-leaf stage for investigation of their responses.

### DNA Isolation and Molecular Analysis of *Pm21* Alleles

Genomic DNA was extracted from leaves of one-leaf-stage plants by the TE-boiling method (He et al., 2017). The marker *MBH1*, developed from the promoter region of *Pm21* gene (Bie et al., 2015b), was used to detect genetic diversity of different *D. villosum* individuals. PCR amplification was carried out according to our previous description (He et al., 2017). PCR products with different sizes were T/A-cloned and sequenced.

### Isolation of *Pm21* Alleles

Total RNA of different *D. villosum* accessions/lines and wheat materials was extracted from seedlings leaves using the TRIzol solution (Life Technologies, Carlsbad, California, USA). About 2 μg of total RNA was used for synthesis of cDNA using the PrimeScript™ II 1st Strand cDNA Synthesis Kit (TaKaRa, Shiga, Japan) according to the manufacturer's guidelines. *Pm21* alleles were isolated from the cDNAs by PCR using the high fidelity PrimeSTAR Max Premix (TaKaRa, Shiga, Japan) and the primer pair (forward primer: 5′-TTACCCGGGCTCACCCGTTGGACTTGGACT-3′; reverse primer: 5′-CCCACTAGTCTCTCTTCGTTACATAATGTAGTGCCT-3′). PCR products were digested with *Sma*I and *Spe*I, inserted into pAHC25-MCS1 and sequenced. The genomic DNA of the alleles in the susceptible materials, DvSus-1 to DvSus-4, DA6V#1, and DA6V#3, were also isolated using PCR with LA *Taq* DNA polymerase (TaKaRa, Shiga, Japan) and the above primer pair. Each *Pm21* allele was amplified from its donor material by three independent PCR, followed by cloning and Sanger sequencing.

### Sequence Data Analysis

Multiple alignment analysis was carried out using the CLUSTAL W tool (Thompson et al., 1994). Nucleotide diversity of *Pm21* alleles and their coding sequences of different domains or non-domain regions was analyzed using the MEGA7 software (Kumar et al., 2016) and assessed by Tajima's test of neutrality (Tajima, 1989). π meant the average number of nucleotide differences per site between two sequences. θ represented Watterson's nucleotide diversity estimator based on the value of π. Synonymous substitution rate (dS), non-synonymous substitution rate (dN), and natural selection for each codon were estimated by the HyPhy program in the MEGA7 software. Sequence logos of LRR motifs were created by the WebLogo tool (Crooks et al., 2004). For evolutionary analyses, all positions containing gaps were eliminated. So, there were a total of 2,718 positions in the final dataset. A phylogenetic tree based on the cDNA sequences of the *Pm21* alleles was constructed using the Neighbor-Joining method in the MEGA7 software (Kumar et al., 2016).

### Accession Numbers

The accession number of *Pm21* gene in the GenBank (https://www.ncbi.nlm.nih.gov/genbank/) is MF370199. The *Pm21* alleles obtained have been deposited in the GenBank under the accession numbers MG831524–MG831526, MG831528–MG831561 and MH184801–MH184806.

## Results

### Powdery Mildew Responses of Different Germplasms

**Figure 1:**
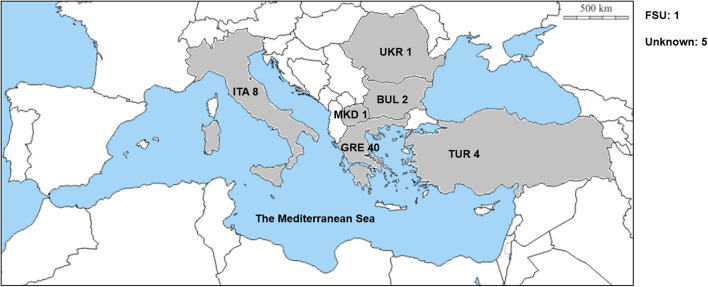
Geographic distribution of the *D. villosum* accessions used in this study. GRE, Greece. ITA, Italy. TUR, Turkey. BUL, Bulgaria. UKR, Ukraine. MKD, Macedonia. FSU, former Soviet Union. Unknown, the origins of the accessions are unclear.

**Figure 2:**
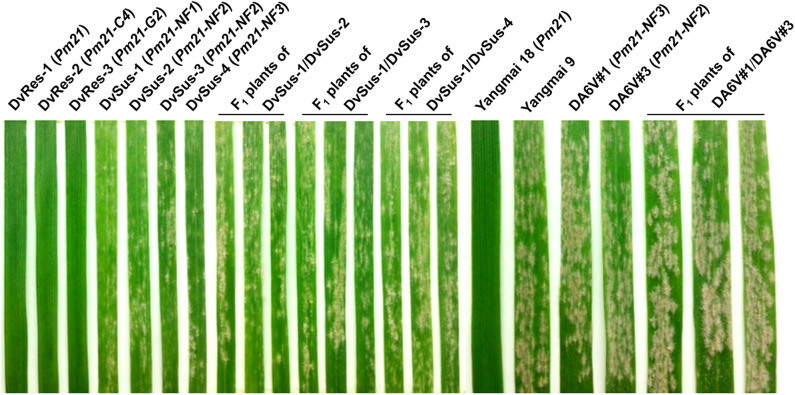
Powdery mildew responses of different *D. villosum* lines, wheat genetic stocks, and F_1_ plants of the crosses DvSus-1/DvSus-2, DvSus-1/DvSus-3, DvSus-1/DvSus-4, Dv6V#1/ Dv6V#3. The plants were inoculated with *Bgt* isolate YZ01 at one-leaf stage. The resistant wheat cv. Yangmai 18 and the susceptible cv. Yangmai 9 were used as the controls. Line DvRes-1 carries *Pm21*. Lines DvRes-2 and DvRes-3, carrying *Pm21-C4* and *Pm21-G2*, were the resistant individuals of the accessions GRA961 and GRA1114, respectively. Lines DvSus-1 to DvSus-4, carrying non-functional *Pm21* genes, were the susceptible individuals in the accessions GRA2738, GRA962, GRA1105, and PI598390, respectively.

The *D. villosum* accessions provided by different germplasm resource institutions were collected from the Mediterranean region, mainly from Greece and Italy (Figure 1; Table S1). A total of 62 accessions were used to detect the responses to *Bgt* isolate YZ01. All plants of the 58 accessions were immune to *Bgt* isolate YZ01, whereas in each of the other four accessions (GRA2738, GRA962, GRA1105, and PI 598390), several individuals (2–5%) were susceptible despite that most plants were resistant. The four susceptible homozygous lines derived from the above accessions were then designated as DvSus-1 to DvSus-4, respectively. The results also showed that the wheat-*D. villosum* chromosome 6V disomic addition lines DA6V#1 and DA6V#3 were susceptible to powdery mildew (Figure 2).

The powdery mildew responses of the F_1_ plants derived from four different crosses, DvSus-1/DvSus-2, DvSus-1/DvSus-3, DvSus-1/DvSus-4, and DA6V#1/DA6V#3, were also assessed. The data showed that all the F_1_ hybrids displayed high susceptibility to *Bgt* isolate YZ01 (Figure 2). It was indicated that there was no obvious allelic complementation in any of the above crosses. Therefore, it was suggested that the potential mutation(s), which led to susceptibility of the four *D. villosum* lines (DvSus-1, DvSus-2, DvSus-3, and DvSus-4) and the two wheat-*D. villosum* chromosome 6V disomic addition lines (DA6V#1 and DA6V#3), may all occur in the alleles of *Pm21*.

### Molecular and Nucleotide Diversity of the *Pm21* Alleles

To understand the diversity at the *Pm21* loci, *MBH1*, designed based on the promoter sequence of *Pm21* (Bie et al., 2015b), was used to detect the resistant individuals from 62 different *D. villosum* accessions. The PCR products were sequenced and eight representative bands with different sizes, 271, 339, 340, 341, 342, 344, 396, and 467 bp, were found. This indicated that insertion-deletion (InDel) polymorphisms exist at the promoter regions of different *Pm21* alleles. Given that all *MBH1* sequences were isolated from resistant individuals, it was suggested that the variations in the promoter regions have no obviously adverse impact on the expression of *Pm21* alleles. In some individuals, two specific DNA bands were observed (Figures S1, S2), suggesting that these individuals might be heterozygous at the *Pm21* loci.

**Figure 3:**
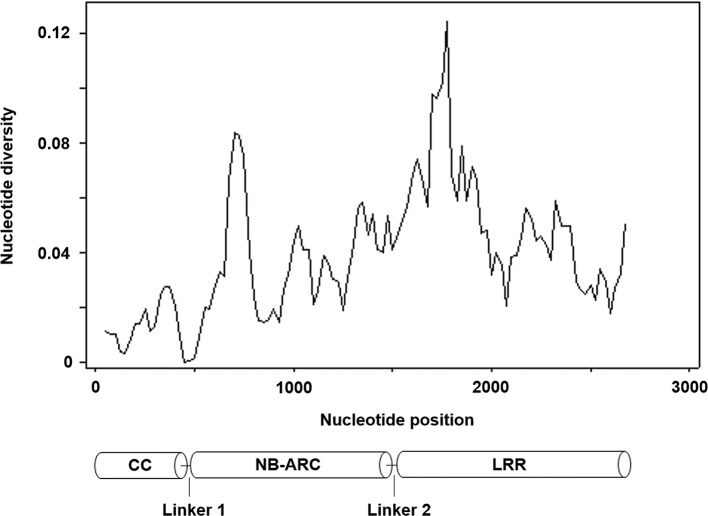
Nucleotide diversity of 38 non-redundant *Pm21* alleles isolated from the resistant *D. villosum* accessions. All the positions containing gaps were eliminated. Therefore, there were a total of 2,718 positions in the final dataset. The predicted protein structure is shown at the bottom.

We then isolated *Pm21* alleles from the resistant individuals of 62 *D. villosum* accessions. Each of the individuals of 52 accessions had one copy of *Pm21* allele. However, due to open pollination of *D. villosum* species, each of the tested individuals of 9 accessions (PI 368886, W619414, W67270, GRA960, GRA1109, GRA1114, GRA2711, GRA2716, and 01C2300013) had two copies of *Pm21* alleles. In addition, three different alleles were, respectively, isolated from three individuals of the accession PI 251478. As a result, a total of 73 *Pm21* alleles were isolated in this study (Table S1). Among them, 38 alleles were non-redundant, sharing 91.7–100% identities with each other. In general, a total of seven InDels (Table S2), including three 3-bp insertions, one 30-bp insertion and three 3-bp deletions, and 400 single nucleotide polymorphism (SNP) sites were identified among these alleles. The 38 non-redundant *Pm21* alleles and their coding sequences of different domains were further used to determine the nucleotide diversity. The average pairwise nucleotide diversity π and Watterson's nucleotide diversity estimator θ of the full-length *Pm21* alleles were 0.039096 and 0.035027, respectively. Compared with the full-length alleles, the values of π and θ of the NB-ARC domain-encoding sequences were slightly lower (π = 0.036868 and θ = 0.034204), whereas those of the CC domain-encoding sequences were significantly lower (π = 0.013115 and θ = 0.012973) and those of the LRR domain-encoding sequences were obviously higher (π = 0.051892 and θ = 0.044652). These results indicated that the CC domain was more conserved than other domains whereas the LRR domain was more variable. We also analyzed the π and θ values of Linker 1 and Linker 2, the regions between the CC and NB-ARC domains, and between the NB-ARC and LRR domains, respectively. The data showed that Linker 1 had no nucleotide diversity. Contrarily, Linker 2 had the highest nucleotide diversity (π = 0.054507 and θ = 0.054092) in different domains or regions of *Pm21* alleles (Figure 3; Table 1). Up to now, the function of Linker 2 is unclear yet. One reasonable explanation for its high variation is that Linker 2 may be an extension of the LRR domain.

**Table 1:** Nucleotide diversity of the *Pm21* alleles and their domains.

**Gene or domain**	**Position (aa)**	**Position (bp)**	***N***	**S**	**π**	**θ**	**D**
FL	1–906	1-2718	2,718	400	0.039096	0.035027	0.437596
CC	1–159	1-477	477	26	0.013115	0.012973	0.037612
Linker 1	160–168	478-504	27	0	0.000000	0.000000	n.a.
NB-ARC	169–502	505-1506	1,002	144	0.036868	0.034204	0.289608
Linker 2	503–524	1507-1572	63	15	0.054507	0.054092	0.024658
LRR	525–906	1573-2718	1,149	215	0.051892	0.044652	0.606923

*FL, full-length Pm21 alleles. Linker 1, linker between the CC and NB-ARC domains. Linker 2, linker between the NB-ARC and LRR domains. n, total number of sites. S, the number of segregating (polymorphic) sites. π, the average number of nucleotide differences per site between two sequences. θ, Watterson's nucleotide diversity estimator based on the value of π. D, Tajima's D statistics for neutrality test. n.a., not applicable*.

### Selection Pressure Analysis

To determine the potential evolutionary selection occurred in *Pm21* alleles, dN and dS rates were assessed using the HyPhy program. The dN/dS ratio of full-length *Pm21*, CC-, NB-ARC-, and LRR-encoding sequences were 0.72046, 0.22671, 0.48723, and 1.15098, respectively, which suggested that the LRR domain might be under positive selection. The dN/dS ratio of the structural LRR residues and the solvent-exposed LRR residues, the two parts of the LRR domain, were 0.88106 and 3.19734, respectively (Table 2). This indicated diversifying selection acting on the solvent-exposed residues in the LRR domain of *Pm21* alleles.

**Table 2:** dN, dS, and dN/dS ratio of *Pm21* alleles and their domains or motifs.

**Gene, domain or motif**	**Position (bp)**	**dN**	**dS**	**dN/dS ratio**
Full-length *Pm21*	1–2,718	0.27309	0.37905	0.72046
CC	1–477	0.08821	0.38906	0.22671
Linker 1	478–504	0.00000	0.00000	n.a.
NB-ARC	505–1,506	0.16928	0.34744	0.48723
Linker 2	1,507–1,572	0.37287	0.86044	0.43336
LRR	1,573–2,718	0.44728	0.38860	1.15098
Structural LRR	-	0.38044	0.43180	0.88106
Solvent-exposed LRR	-	0.69844	0.21844	3.19734
LRR1	1,573–1,635	0.27554	0.83026	0.33187
LRR2	1,636–1,707	0.33048	0.70341	0.46982
LRR3	1,708–1,785	0.71357	0.69042	1.03357
LRR4	1,786–1,851	0.46933	0.18153	2.58544
LRR5	1,852–1,917	0.62027	0.34232	1.81196
LRR6	1,918–1,980	0.29715	0.11953	2.48594
LRR7	1,981–2,049	0.41649	0.24384	1.70807
LRR8	2,050–2,127	0.19744	0.25129	0.78572
LRR9	2,128–2,208	0.43540	0.66865	0.65117
LRR10	2,209–2,277	0.53271	0.19266	2.76496
LRR11	2,278–2,349	0.48589	0.05661	8.58259
LRR12	2,350–2,418	0.46961	0.67420	0.69654
LRR13	2,421–2,487	0.26525	0.27089	0.97917
LRR14	2,488–2,562	0.19280	0.78675	0.24506
LRR15	2,563–2,652	0.29745	0.14091	2.11088
LRR16	2,653–2,718	1.26260	0.00000	Infinite

*FL, full-length Pm21 alleles. Linker 1, the linker between the CC and NB-ARC domains. Linker 2, the linker between the NB-ARC and LRR domains. Solvent-exposed LRR, the residue x in the LxxLxLxx motif. Structural LRR, other residues except the residue x in the LRR domain (Srichumpa et al., 2005). LRR1 to LRR16, 16 LRR motifs predicted in the LRR domain. n.a., not applicable*.

**Figure 4:**
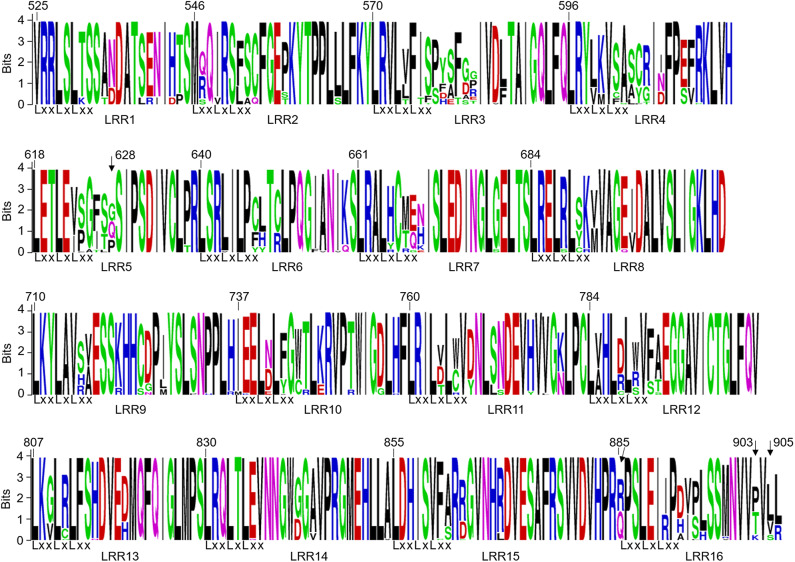
Sequence logos of 16 LRR motifs encoded by *Pm21* alleles. In the LxxLxLxx motifs, x represents the predicted solvent-exposed LRR residues, and L represents a leucine or another aliphatic amino acid residue. The sites at positions 628, 885, 903, and 905 pointed by arrows are predicted to be under positive selection.

The LRR domain of *Pm21* consists of 16 LRR motifs. The dN/dS ratios of 8 LRR motifs (LRR4-LRR7, LRR10, LRR11, LRR15, and LRR16) were greater than 1. Among them, the dN/dS ratio of LRR11 was 8.58259 and that of LRR16 was infinite because its dS value was zero (Figure 4; Table 2). These results indicated that the above 8 LRR motifs have undergone positive selection. In the LRR domain, four sites at the positions 628, 885, 903, and 905 were subject to positive selection, detected by four different models (Felsensten 1981 model, Hasegawa-kishino-Yano model, Tamura-Nei model, and General Time Reversible model). Position 628 lied in the LRR5 motif and positions 885, 903, and 905 were all located in the LRR16 motif (Figure 4; Table S3).

### Phylogenetic Analysis and Classification of the *Pm21* Alleles

**Figure 5:**
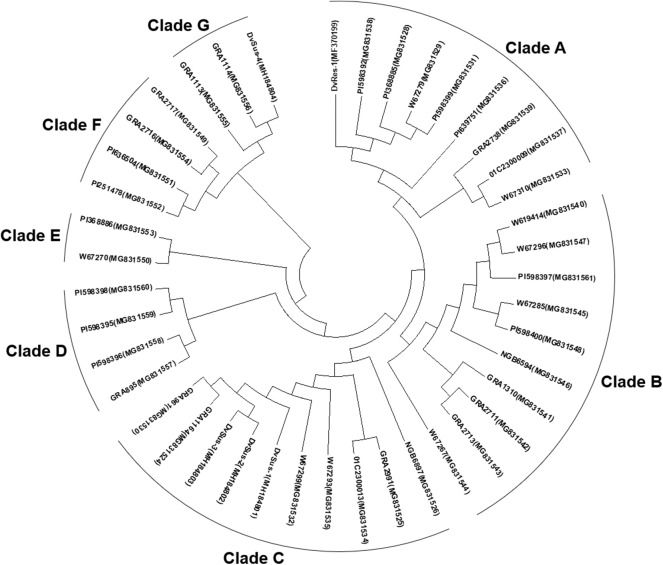
Phylogenetic tree of *Pm21* alleles constructed by the Neighbor-Joining method. The GenBank accession numbers are shown in brackets.

The phylogenetic tree for *Pm21* alleles showed that 38 non-redundant *Pm21* alleles were clustered into seven clades (Clade A to G). Among these clades, Clades A, B, and C were the major types in the *D. villosum* populations, which included 26 members, accounting for 68.4% (Figure 5).

According to the clades categorized in the phylogenetic tree, the *Pm21* alleles isolated from the resistant *D. villosum* accessions were correspondingly divided into seven classes (Class A to G). Class A consisted of 9 alleles, *Pm21-A1* to *Pm21-A9*, whose open reading frames (ORFs) were 2,730 bp in length sharing the highest identities with *Pm21* (99.2% on average). Class B contained 10 alleles, *Pm21-B1* to *Pm21-B10*, most of which were 2,724 bp sharing 96.6% identity with *Pm21* on average. Class C harbored 7 alleles, *Pm21-C1* to *Pm21-C7*, with 2,730 bp in length and had 96.7% identity with *Pm21* on average. The remaining 12 alleles, sharing 92.1–97.0% identities with *Pm21*, were divided into four classes, Class D to G, whose obvious sequence characteristics was a 30-bp insertion compared with *Pm21* (Table 3).

**Table 3:** Classification of *Pm21* alleles isolated from resistant individuals of *D. villosum*.

**Class**	**Allele**	**GenBank accession** **number**	**ORF length** **(bp)**	**InDel** **(compared with *Pm21*)**	**Identity with (%)**		**Occurrence in** **population**
					***Pm21* on average**	**Class**	
A	*Pm21* (*Pm21-A1*)	MF370199	2,730	-	99.2	98.0–100	7
	*Pm21-A2*	MG831538	2,730	-			1
	*Pm21-A3*	MG831528	2,730	-			2
	*Pm21-A4*	MG831529	2,730	-			1
	*Pm21-A5*	MG831531	2,730	-			1
	*Pm21-A6*	MG831536	2,730	-			1
	*Pm21-A7*	MG831539	2,730	-			1
	*Pm21-A8*	MG831537	2,730	-			1
	*Pm21-A9*	MG831533	2,730	-			1
B	*Pm21-B1*	MG831540	2,724	In-2, Del-1—Del-3	96.6	97.4–100	1
	*Pm21-B2*	MG831545	2,724	In-2, Del-1—Del-3			5
	*Pm21-B3*	MG831546	2,724	In-2, Del-1—Del-3			1
	*Pm21-B4*	MG831547	2,724	In-2, Del-1—Del-3			4
	*Pm21-B5*	MG831548	2,724	In-2, Del-1—Del-3			1
	*Pm21-B6*	MG831561	2,727	In-2, Del-1—Del-2			2
	*Pm21-B7*	MG831541	2,724	Del-2—Del-3			4
	*Pm21-B8*	MG831542	2,724	Del-2—Del-3			1
	*Pm21-B9*	MG831543	2,724	Del-2—Del-3			1
	*Pm21-B10*	MG831544	2,724	Del-2—Del-3			3
C	*Pm21-C1*	MG831524	2,730	-	96.7	97.9–99.9	4
	*Pm21-C2*	MG831525	2,730	-			1
	*Pm21-C3*	MG831526	2,730	-			4
	*Pm21-C4*	MG831530	2,730	-			2
	*Pm21-C5*	MG831532	2,730	-			1
	*Pm21-C6*	MG831534	2,730	-			1
	*Pm21-C7*	MG831535	2,730	-			1
D	*Pm21-D1*	MG831557	2,766	In-1–In-3	92.4	99.2–100	3
	*Pm21-D2*	MG831558	2,766	In-1–In-3			1
	*Pm21-D3*	MG831559	2,766	In-1–In-3			1
	*Pm21-D4*	MG831560	2,766	In-1–In-3			1
E	*Pm21-E1*	MG831550	2,760	In-1–In-2, Del-1	96.4	97.8	1
	*Pm21-E2*	MG831553	2,760	In-1–In-2, Del-1			1
F	*Pm21-F1*	MG831549	2,760	In-1–In-2, Del-1	95.8	99.3–100	4
	*Pm21-F2*	MG831551	2,760	In-1–In-2, Del-1			2
	*Pm21-F3*	MG831552	2,760	In-1–In-2, Del-1			1
	*Pm21-F4*	MG831554	2,760	In-1–In-2, Del-1			1
G	*Pm21-G1*	MG831555	2,763	In-1, In-4, Del-1	95.2	99.8	3
	*Pm21-G2*	MG831556	2,763	In-1, In-4, Del-1			1

### Natural Variations of *Pm21* Alleles in Susceptible Germplasms

To test the rare natural variations leading to lose of resistance to powdery mildew, we isolated *Pm21* alleles from the susceptible *D. villosum* lines DvSus-1 to DvSus-4, derived from the accessions GRA2738, GRA962, GRA1105, and PI 598390, respectively. The non-functional allele *Pm21-NF1* isolated from the genome of DvSus-1 was 3,699 bp in length, whose ORF was 2,730 bp. Compared with *Pm21, Pm21-NF1* had 98 SNPs; however, compared with the 38 non-redundant alleles isolated from the resistant *D. villosum* accessions, *Pm21-NF1* only had two specific variations. The first variation was a transversion G61T leading to the amino acid change A21S in the CC domain. The second variation was a transition A821G resulting in the change D274G (Figure S3A), corresponding to the latter aspartate (D) in kinase-2 motif (also called Walker B motif; consensus sequence: LLVLDDVW) in the NB-ARC domain. The latter D is considered to act as the catalytic site for ATP hydrolysis and activation of disease resistance protein (Meyers et al., 1999; Tameling et al., 2006). Here, bioinformatic analysis showed that the latter D was highly conserved in all the tested disease resistance proteins from *Arabidopsis thaliana*, barley (*Hordeum vulgare* L.) and wheat (Figure S4), suggesting that the amino acid change D274G might lead to loss-of-function of *Pm21-NF1*.

The genomic sequence of the non-functional allele *Pm21-NF2* isolated from the susceptible DvSus-2 was 3,698 bp in length, whose ORF contained a 1-bp deletion after position 876, leading to frame shift and resulting in a truncated protein (296 aa). The variations of *Pm21* alleles isolated from DvSus-3 and DA6V#3 were both identical to that of *Pm21-NF2*. In DvSus-4 and DA6V#1, the sequences of the alleles were identical (4,988 bp) and designated as *Pm21-NF3*. *Pm21-NF3* harbored an insertion of 1281 bp that caused a premature stop codon (Figure S3B) and led to loss of the last four LRR motifs. These results suggested that the non-functional *Pm21* alleles in DA6V#1 and DA6V#3 both directly originated from their *D. villosum* donors susceptible to powdery mildew.

### Molecular Tracing of the Origins of Non-functional *Pm21* Alleles

**Figure 6:**
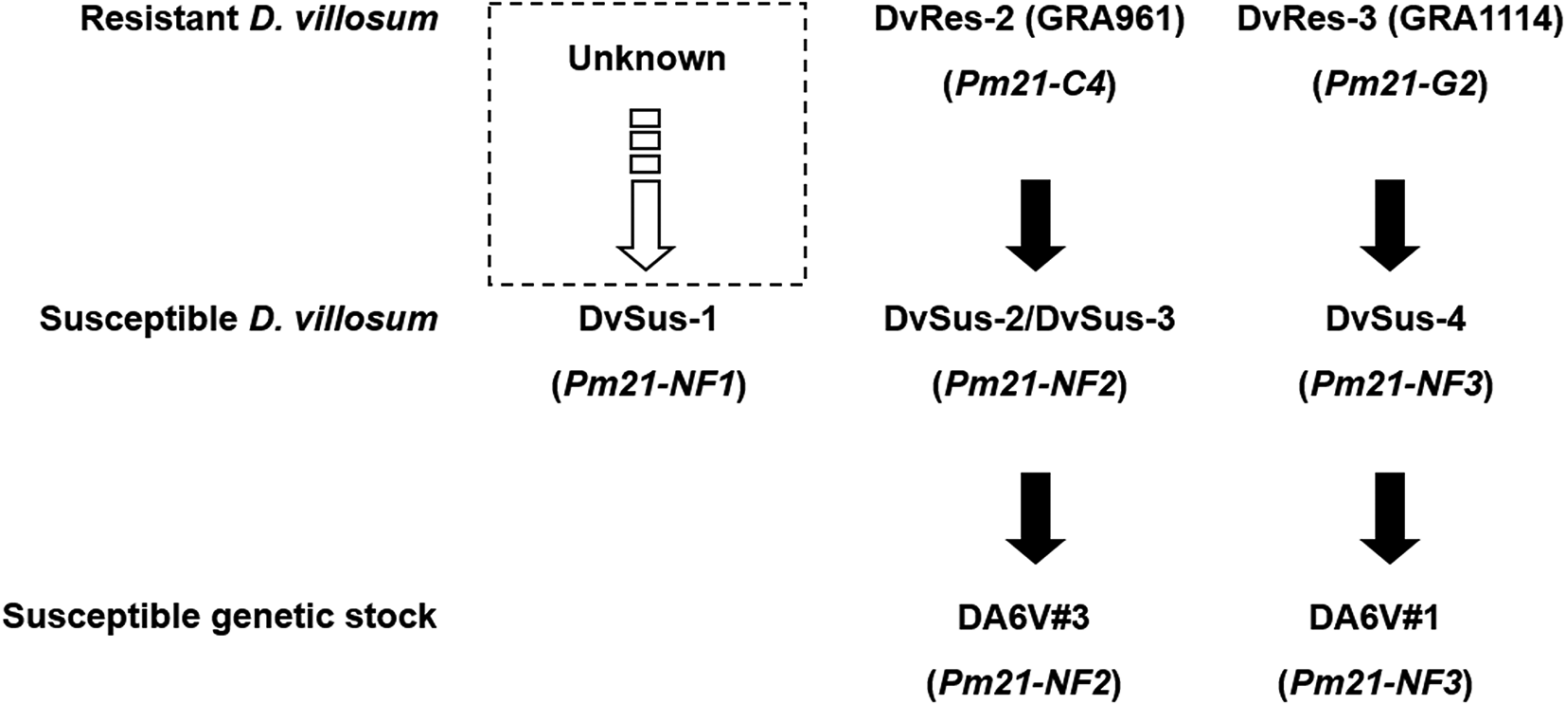
Origins of the non-functional alleles *Pm21-NF1* to *Pm21-NF3*. Among them, the origin of *Pm21-NF1* is unclear yet.

Phylogenetic analysis showed that DvSus-1, DvSus-2, DvSus-3, GRA961, and GRA1164 were clustered in Clade C (Figure 5). In contrast to the alleles, *Pm21-C4* in GRA961 and *Pm21-C1* in GRA1164, the non-functional allele *Pm21-NF1* in DvSus-1 had 8 and 10 SNPs, and *Pm21-NF2* in DvSus2/DvSus-3 had 1 and 3 SNPs, respectively (Figure S3B). This suggested that the non-functional allele *Pm21-NF2* originated from the allele *Pm21-C4* in the resistant accession GRA961 (Figures 5, 6; Table 3). In the tested accessions, the origin of *Pm21-NF1* could not be well-traced yet.

The data also indicated that lines DvSus-4, GRA1113, and GRA1114 were clustered in Clade G (Figure 5). Except the 1281-bp insertion, *Pm21-NF3* in DvSus-4 had no difference from *Pm21-G2* in GRA1114 (Figure S3C). This result revealed that the non-functional allele *Pm21-NF3* came from the variation of the allele *Pm21-G2* in the resistant accession GRA1114 (Figures 5, 6; Table 3).

## Discussion

### Diversity, Classification and Geographic Distribution of *Pm21* Alleles

As a wild relative of wheat, *D. villosum* possesses several powdery mildew resistance genes that have important potential for controlling wheat powdery mildew disease (He et al., 2017). Among them, *Pm21* and *PmV*, located on chromosome 6VS derived from different *D. villosum* accessions, confer powdery mildew resistance at whole-plant growth stages. It seems that *Pm21* and *PmV* may be allelic (Bie et al., 2015b). Both *Pm55* and *Pm62* confer resistance at adult-plant stage but not at the seedling stage (Zhang et al., 2016, 2018). In this study, *Bgt*-responses of all *D. villosum* accessions were detected at one-leaf stage, which could exclude the resistance conferred by *Pm55* and *Pm62*. Therefore, the seedling-resistance in these materials was considered to be provided by *Pm21* alleles.

Recently, the broad-spectrum powdery mildew resistance gene *Pm21* was isolated from *D. villosum* using the map-based cloning strategy (He et al., 2018). Based on the investigation of powdery mildew responses of different *D. villosum* accessions collected from the Mediterranean countries, we isolated 73 *Pm21*-like sequences from the resistant individuals. The previous work showed that *Pm21* is adjacent to another CC-NBS-LRR-encoding gene *DvRGA1* (He et al., 2018). Although *DvRGA1* is the highest matched gene of *Pm21* in Genbank database, they had only 72.7% nucleotide sequence identity. Here, the isolated *Pm21*-like genes shared 91.7–100% identities with each other, indicating that all the sequences are identical or allelic to *Pm21*. Of the 73 sequences, 38 were different from each other. Compared with *Pm21*, the other 37 non-redundant alleles have seven InDels involved in 3-bp, 6-bp, 30-bp, 33-bp, or 36-bp, which make the alleles maintain correct ORFs and encode full-length proteins. The alleles also had many SNPs and the average pairwise nucleotide diversity of the LRR-encoding region was significantly higher than those of the CC- or NB-ARC-encoding regions. Compared with other domains, the LRR domain were supposed to have undergone faster evolution. Because all of the individuals containing these alleles were still effective against the highly virulent *Bgt* isolate YZ01, it was proposed that the wide variations of *Pm21* alleles have no obviously adverse effect on the disease resistance. However, whether they still keep broad-spectrum resistance remains to be disclosed.

Phylogenetic analysis identified seven independent clades that involved all the *Pm21* alleles. Among them, Classes A to C represented the three major classes. The functional *Pm21* gene was originally found in an accession provided by Cambridge Botanic Garden in the United Kingdom, but the exact collection site of this accession was unclear. *Pm21*, with the systemic name *Pm21-A1* here, belongs to Class A whose members were only found in the accessions of Greece or Turkey. In particular, among the six isolated sequences identical to *Pm21*, five came from independent Greece accessions and one from a Turkey accession. Therefore, based on the present data, it was proposed that the original *D. villosum* donor of *Pm21* might come from Greece or Turkey.

Geographic distributions of different *Pm21* alleles were further investigated in this study. It is indicated that the *Pm21* alleles isolated from Greece *D. villosum* accessions had more genetic diversity and covered the most members of all the seven classes (Class A to G). In addition, *Pm21-A8, Pm21-E2*, and *Pm21-F3* were only detected in Turkey accessions, and *Pm21-B7* and *Pm21-G2* were only detected in Italy accessions (Table S1). The characteristics of geographic distributions of the *Pm21* alleles may help to search the accessions carrying specific *Pm21* alleles as donors for future breeding purpose.

### Variations and Origins of Non-functional *Pm21* Alleles in Susceptible *D. villosum* Lines and Wheat Genetic Stocks

It has been believed that *D. villosum* resources are all resistant to wheat powdery mildew (Qi et al., 1998). In our previous work, four *D. villosum* lines DvSus-1 to DvSus-4 susceptible to powdery mildew were identified from different accessions of *D. villosum*, which made it possible to clone *Pm21* using the map-based cloning strategy (He et al., 2017, 2018). In this study, we demonstrated that the variations of *Pm21* alleles, *Pm21-NF1* to *Pm21-NF3*, isolated from the four susceptible *D. villosum* lines, involved point mutation, deletion and insertion, respectively. Among them, *Pm21-NF1* had an important amino acid change (D274G) in the highly conserved kinase-2 motif of the NB-ARC domain that might hamper the function of ATP hydrolysis (Meyers et al., 1999; Tameling et al., 2006), while *Pm21-NF2* and *Pm21-NF3* both encoded truncated proteins caused by premature stop codons.

Previously, the wheat-*D. villosum* chromosome 6V disomic addition lines DA6V#1 and DA6V#3 were reported to be highly susceptible to powdery mildew (Qi et al., 1998; Liu et al., 2011). During the creation of the two addition lines, colchicine was used for chromosome doubling, which is proved to be an effective mutagen in fact (Gilbert and Patterson, 1965). So, researchers did not know if the susceptibilities of DA6V#1 and DA6V#3 came from colchicine treatment or the *D. villosum* donors. Since *Pm21* has been cloned, through sequencing of allele genes here, we demonstrated that *Pm21* alleles isolated from DA6V#1 and DA6V#3 had identical variations to *Pm21-NF3* (DvSus-4) and *Pm21-NF2* (DvSus-2 and DvSus-3), respectively. Therefore, it was suggested that the variations of the *Pm21* alleles from DA6V#1 and DA6V#3 both originated from their *D. villosum* donors, rather than colchicine treatment.

The non-functional alleles, *Pm21-NF1, Pm21-NF2*, and *Pm21-NF3*, were found in the accessions GRA2738, GRA962, PI 598390, respectively. In theory, their wild-type genes could be isolated from the corresponding accessions above. We tried to do so but not succeeded. The major reason may be that *D. villosum* is highly outcrossing which causes that the pollen with a mutated gene is subject to separate from the one carrying a corresponding wild-type gene. Therefore, we attempted to trace the origins of the non-functional alleles through evolutionary analysis. The origins of the two non-functional alleles, *Pm21-NF2* and *Pm21-NF3*, were both traceable in the natural populations of *D. villosum*. Except the identified mutations, the sequences of *Pm21-NF2* and *Pm21-NF3* were entirely identical to those of *Pm21-C4* and *Pm21-G2* that were cloned from the resistant individuals of the accessions GRA961 and GRA1114, respectively. Hence, we concluded that the non-functional alleles *Pm21-NF2* and *Pm21-NF3* originated from *Pm21-C4* and *Pm21-G2*, respectively. However, the origin of *Pm21-NF1* remains unclear yet.

### Diversifying Selection Acting on the Solvent-Exposed LRR Residues of *Pm21* Alleles

It was confirmed that the broad-spectrum resistance of *Pm21* is conferred by a single CC-NBS-LRR-encoding gene (He et al., 2018). However, it is believed that the resistance provided by such kind of genes is most likely race-specific, which is prone to be overcome by fast-evolving pathogens. For instance, *Pm8* from rye (*Secale cereale* L.), also encoding a CC-NBS-LRR protein, previously provided effective resistance to wheat powdery mildew (Hurni et al., 2013), has lost its resistance in most wheat producing regions with the worldwide utilization. In this study, the value of dN/dS (3.19734) significantly exceeded 1 in the solvent-exposed LRR residues, which is considered to take part in the specific recognition of pathogens (Meyers et al., 1999). This result suggested that the solvent-exposed LRR residues of *Pm21* have been undergone diversifying selection and may play critical roles in resistance specificity. This situation is similar to those of race-specific powdery mildew resistance gene *Pm3* from wheat (Srichumpa et al., 2005) and *Mla* from barley (Seeholzer et al., 2010). In several works, the researchers reported that the wheat varieties carrying *Pm21* could be infected by *Bgt* pathogens in different regions (Shi et al., 2009; Yang et al., 2009). Therefore, combined the data given by evolutionary analysis, it is speculated that *Pm21* may be a race-specific resistance gene although it still provides broad-spectrum resistance to the most *Bgt* isolates so far.

Since 1995 when the translocation line of wheat-*D. villosum* T6AL.6VS was released, many wheat varieties carrying *Pm21* have been commercialized in China, mainly in the middle and lower reaches of the Yangtze River Valley and the southwest wheat-producing regions, where *Bgt* pathogen is prevailing (Jiang et al., 2014; Bie et al., 2015a; Cheng et al., 2020). The long-time and wide-range application of *Pm21* in agriculture would accelerate the evolution of *Bgt* pathogens. Correspondingly, *Pm21* would face to an increasing risk of losing its resistance to powdery mildew. Consequently, it will be a great challenge to sustainably utilize the *Pm21* resistance in the future. In this study, a total of 38 non-redundant *Pm21* alleles were obtained, which allows to comparatively analyze their fine functions against *Bgt* pathogens in further researches. Utilization of different *Pm21* alleles with functional diversity would be a way to extend the lifespan of *Pm21* resistance in wheat production. The marker *MBH1*, which can reveal genetic diversity of *Pm21* alleles in some degree, will be a useful tool when transferring them from *D. villosum* into common wheat. Other reasonable means would be diversifying use of *Pm* genes in field, such as pyramiding other effective *Pm* gene(s) into *Pm21*-carrying varieties or exploring new *Pm* genes and developing wheat varieties carrying different *Pm* genes.

## Data Availability Statement

The datasets generated for this study can be found in the GenBank, MG831524–MG831526, MG831528–MG831561, MH184801–MH184806.

## Author Contributions

HH, CL, and SZ conceived and designed the experiments. HH, JJ, JT, YF, XW, RH, and TB performed the experiments. HH, JJ, and HL analyzed the data and wrote the paper.

## Conflict of Interest

The authors declare that the research was conducted in the absence of any commercial or financial relationships that could be construed as a potential conflict of interest.

## Supplementary Material

Figure S1 Molecular analysis of the diversity of *D. villosum* by the marker *MBH1* that was developed from the promoter region of *Pm21*. M, DNA marker DL2000. Line 1 to 24, PCR products obtained from resistant individuals of different *D. villosum* accessions.

Figure S2 Multiple sequence alignment of different representative products PCR-amplified with the marker *MBH1*.

Figure S3 Detection of mutations in the non-functional *Pm21* alleles. **(A)** Mutations of *Pm21-NF1* in DvSus-1 contrasted to *Pm21*. **(B)** Mutations of *Pm21-NF2* in DvSus-2, DvSus-3, and DA6V#3 contrasted to *Pm21-C4* in DvRes-2 (derived from GRA961). **(C)** Mutations of *Pm21-NF3* in DvSus-4 and DA6V#1 in contrast to *Pm21-G2* in DvRes-3 (derived from GRA1114). SNPs, tandem premature stop codons and insertion sequences are shown by arrows, underlines and brackets, respectively.

Figure S4 Multiple sequence alignment of the surrounding sequences of kinase-2 motif (consensus sequence: LLVLDDVW) of plant disease resistance proteins. The conserved second aspartate (D) of kinase-2 motif is marked by an arrow.

Table S1 *Pm21* alleles and the corresponding germplasms.

Table S2 InDel polymorphisms in the *Pm21* alleles. All InDels, compared with *Pm21*, occur after the positions showed in the brackets.

Table S3 Amino acid sites under positive selection.
