# Supplementary figures and images for: Genetic Diversity and Evolutionary Analyses Reveal the Powdery Mildew Resistance Gene Pm21 Undergoing Diversifying Selection
Source: Front Genet. 2020 May 12;11:489. doi: 10.3389/fgene.2020.00489 (PMC7241504; doi:10.3389/fgene.2020.00489)

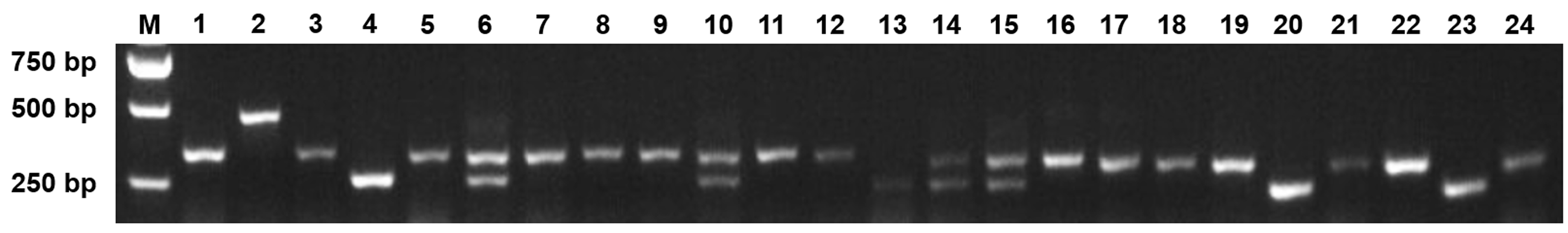

Supplement: Figure S1 — Molecular analysis of the diversity of D. villosum by the marker MBH1 that was developed from the promoter region of Pm21. M, DNA marker DL2000. Line 1 to 24, PCR products obtained from resistant individuals of different D. villosum accessions. [file Image_1.TIF]

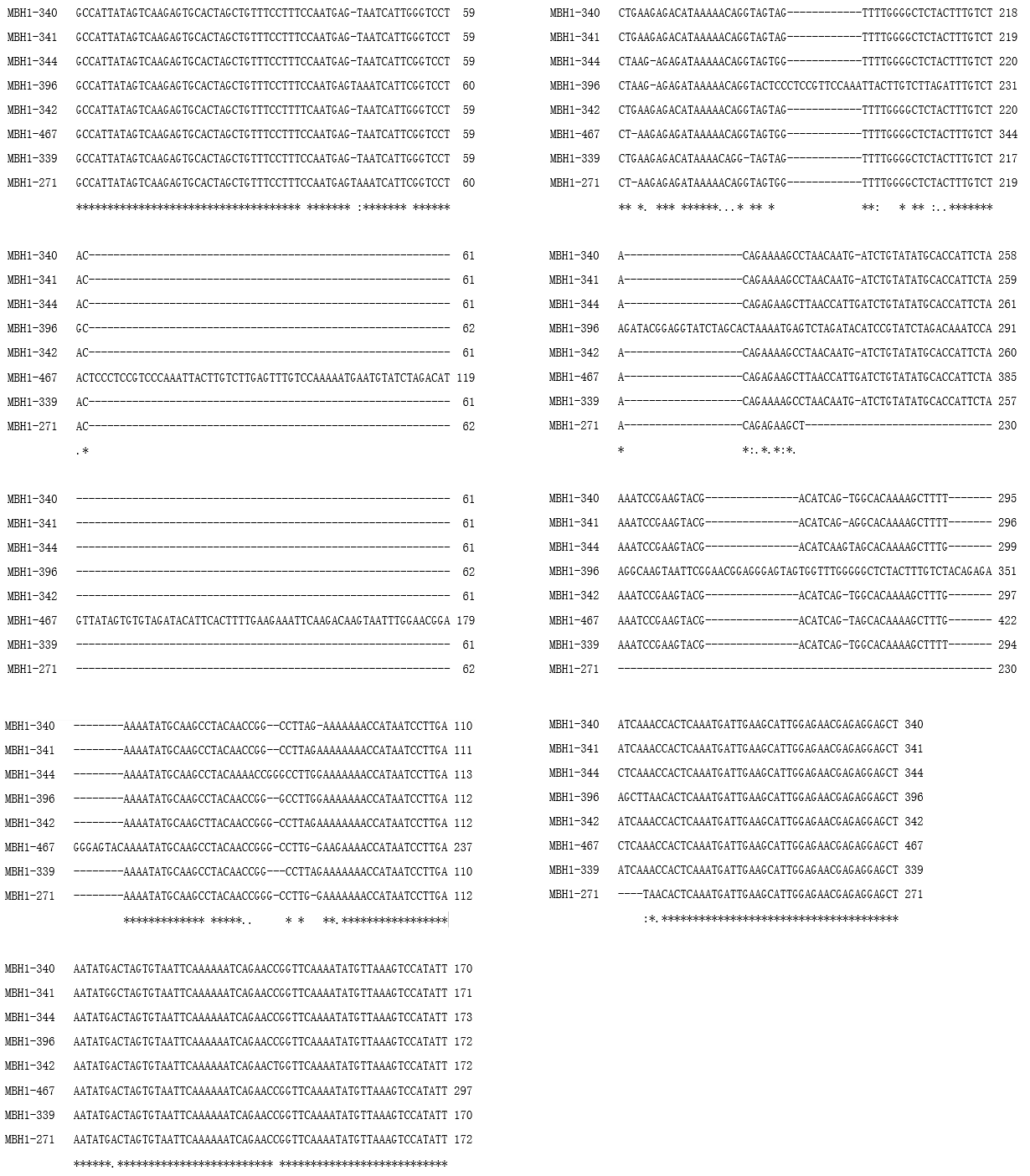

Supplement: Figure S2 — Multiple sequence alignment of different representative products PCR-amplified with the marker MBH1. [file Image_2.TIF]

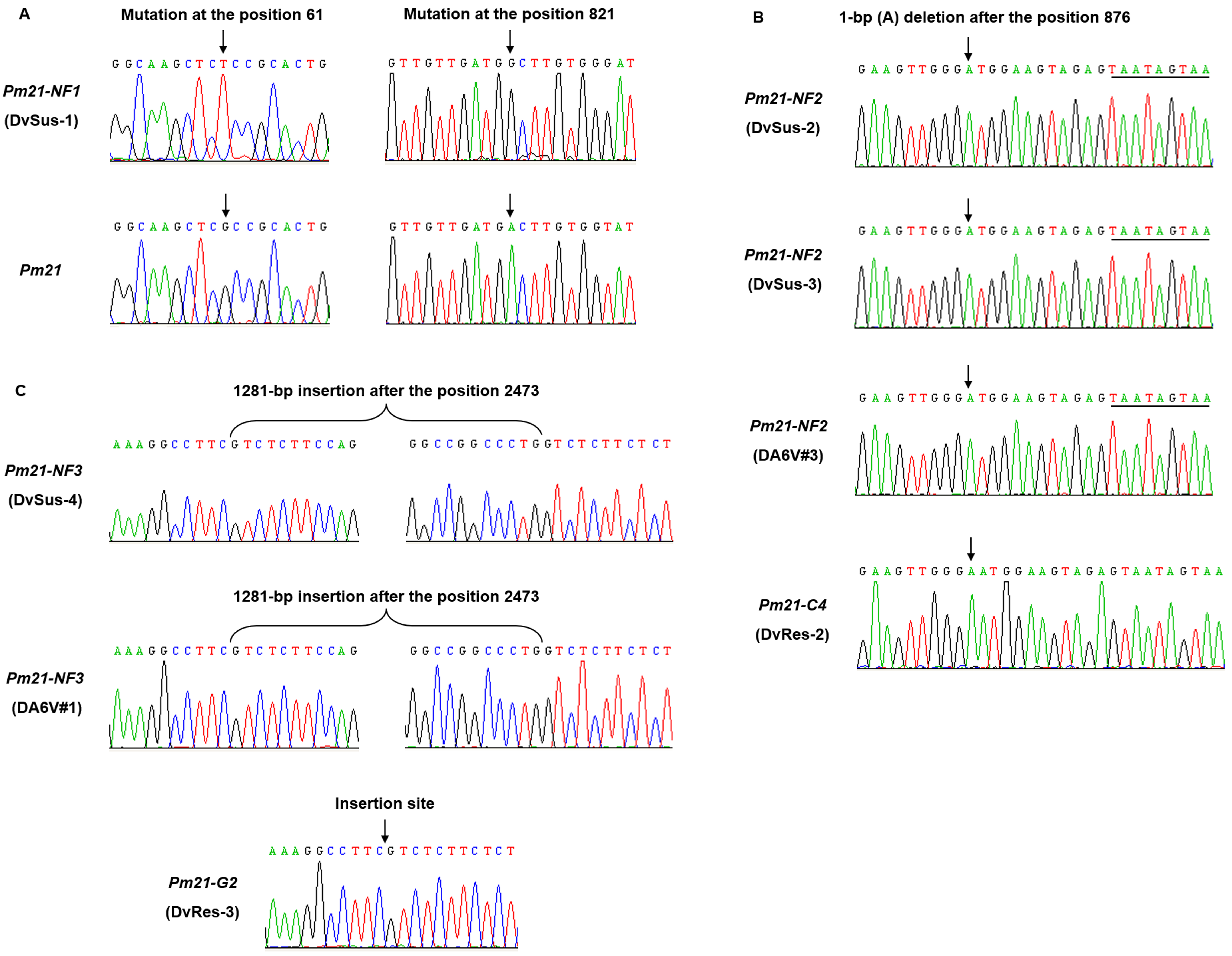

Supplement: Figure S3 — Detection of mutations in the non-functional Pm21 alleles. (A) Mutations of Pm21-NF1 in DvSus-1 contrasted to Pm21. (B) Mutations of Pm21-NF2 in DvSus-2, DvSus-3, and DA6V#3 contrasted to Pm21-C4 in DvRes-2 (derived from GRA961). (C) Mutations of Pm21-NF3 in DvSus-4 and DA6V#1 in contrast to Pm21-G2 in DvRes-3 (derived from GRA1114). SNPs, tandem premature stop codons and insertion sequences are shown by arrows, underlines and brackets, respectively. [file Image_3.TIF]

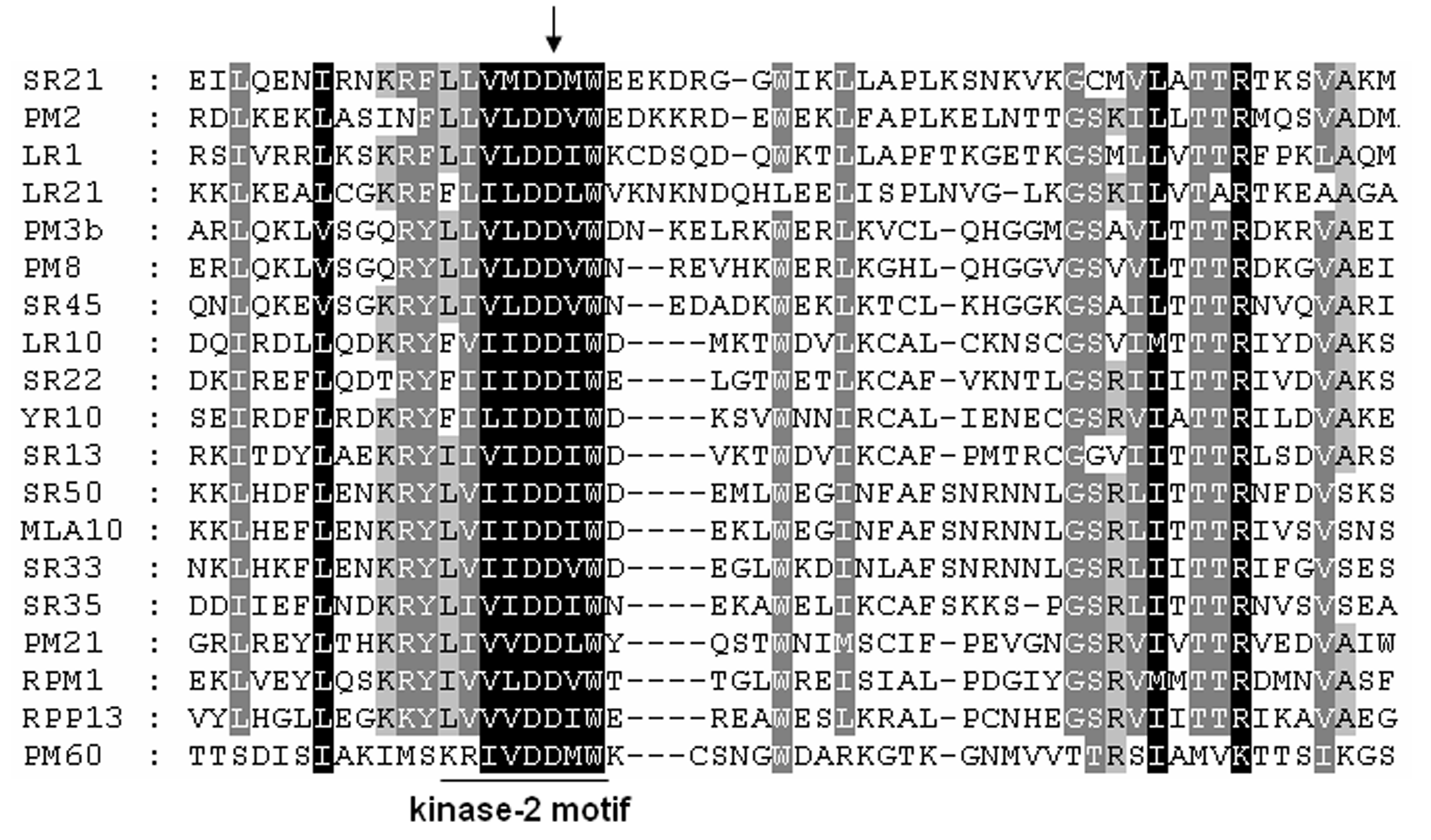

Supplement: Figure S4 — Multiple sequence alignment of the surrounding sequences of kinase-2 motif (consensus sequence: LLVLDDVW) of plant disease resistance proteins. The conserved second aspartate (D) of kinase-2 motif is marked by an arrow. [file Image_4.TIF]
