# Supplementary material for: Genetic Diversity and Evolutionary Analyses Reveal the Powdery Mildew Resistance Gene Pm21 Undergoing Diversifying Selection
Source: Front Genet. 2020 May 12;11:489. doi: 10.3389/fgene.2020.00489 (PMC7241504; doi:10.3389/fgene.2020.00489)
Supplement: Table S1 — Pm21 alleles and the corresponding germplasms. [file Table_1.DOCX]

**Table S1.** *Pm21* alleles and the corresponding germplasms

| **Allele** | **GenBank accession number** | **Sequence obtained** | **Original accession** | **Origin** | **Provider** |
| --- | --- | --- | --- | --- | --- |
| *Pm21* | MF370199 | Seq-R1^a^ | Unknown | Unknown | CBG |
| (*Pm21-A1*) |  | Seq-R2^a^ | PI 598393 | Greece | GRIN |
|  |  | Seq-R3^a^ | W67264 | Greece | GRIN |
|  |  | Seq-R4^a^ | W67280 | Greece | GRIN |
|  |  | Seq-R5^a^ | W67266 | Greece | GRIN |
|  |  | Seq-R6^a^ | PI 598391 | Greece | GRIN |
|  |  | Seq-R7^a^ | PI 251478* | Turkey | GRIN |
| *Pm21-A2* | MG831538 | Seq-R8 | PI 598392 | Greece | GRIN |
| *Pm21-A3* | MG831528 | Seq-R9^b^ | PI 368885 | Turkey | GRIN |
|  |  | Seq-R10^b^ | PI 598394 | Greece | GRIN |
| *Pm21-A4* | MG831529 | Seq-R11 | W67279 | Greece | GRIN |
| *Pm21-A5* | MG831531 | Seq-R12 | PI 598399 | Greece | GRIN |
| *Pm21-A6* | MG831536 | Seq-R13 | PI 639751 | Greece | GRIN |
| *Pm21-A7* | MG831539 | Seq-R14 | GRA2738** | Greece | GBIS-IPK |
| *Pm21-A8* | MG831537 | Seq-R15 | 01C2300009 | Turkey | GRIN-Czech |
| *Pm21-A9* | MG831533 | Seq-R16 | W67310 | Greece | GRIN |
| *Pm21-B1* | MG831540 | Seq-R17 | W619414* | Bulgaria | GRIN |
| *Pm21-B2* | MG831545 | Seq-R18^c^ | W67285 | Greece | GRIN |
|  |  | Seq-R19^c^ | W67286 | Greece | GRIN |
|  |  | Seq-R20^c^ | 01C2300010 | Greece | GRIN-Czech |
|  |  | Seq-R21^c^ | 01C2300014 | Macedonia | GRIN-Czech |
|  |  | Seq-R22^c^ | 01C2300004 | Bulgaria | GRIN-Czech |
| *Pm21-B3* | MG831546 | Seq-R23 | NGB6594 | Greece | NordGen |
| *Pm21-B4* | MG831547 | Seq-R24^d^ | W67296 | Greece | GRIN |
|  |  | Seq-R25^d^ | W67270* | Greece | GRIN |
|  |  | Seq-R26^d^ | PI 368886* | Turkey | GRIN |
|  |  | Seq-R27^d^ | 01C2300013* | Greece | GRIN-Czech |
| *Pm21-B5* | MG831548 | Seq-R28 | PI 598400 | Greece | GRIN |
| *Pm21-B6* | MG831561 | Seq-R29^e^ | PI 598397 | Greece | GRIN |
|  |  | Seq-R30^e^ | W619414* | Bulgaria | GRIN |
| *Pm21-B7* | MG831541 | Seq-R31^f^ | GRA1310 | Italy | GBIS-IPK |
|  |  | Seq-R32^f^ | GRA1114* | Italy | GBIS-IPK |
|  |  | Seq-R33^f^ | GRA1109* | Italy | GBIS-IPK |
|  |  | Seq-R34^f^ | GRA960* | Italy | GBIS-IPK |
| *Pm21-B8* | MG831542 | Seq-R35 | GRA2711* | Greece | GBIS-IPK |
| *Pm21-B9* | MG831543 | Seq-R36 | GRA2713 | Greece | GBIS-IPK |
| *Pm21-B10* | MG831544 | Seq-R37^g^ | W67267 | Greece | GRIN |
|  |  | Seq-R38^g^ | GRA992 | Unknown | GBIS-IPK |
|  |  | Seq-R39^g^ | NGB6896 | Ukraine | NordGen |
| *Pm21-C1* | MG831524 | Seq-R40^h^ | GRA1164 | Italy | GBIS-IPK |
| Table S1 (continued) | | | | | |
| **Allele** | **GenBank accession number** | **Sequence obtained** | **Original accession** | **Origin** | **Provider** |
|  |  | Seq-R41^h^ | NGB7448 | Greece | NordGen |
|  |  | Seq-R42^h^ | GRA960* | Italy | GBIS-IPK |
|  |  | Seq-R43^h^ | GRA1105** | Italy | GBIS-IPK |
| *Pm21-C2* | MG831525 | Seq-R44 | GRA2991 | Greece | GBIS-IPK |
| *Pm21-C3* | MG831526 | Seq-R45^i^ | NGB6897 | Greece | NordGen |
|  |  | Seq-R46^i^ | W67302 | Greece | GRIN |
|  |  | Seq-R47^i^ | W67289 | Greece | GRIN |
|  |  | Seq-R48^i^ | PI 598390** | Former Soviet Union | GRIN |
| *Pm21-C4* | MG831530 | Seq-R49^j^ | GRA961 | Unknown | GBIS-IPK |
|  |  | Seq-R50^j^ | PI 251478* | Turkey | GRIN |
| *Pm21-C5* | MG831532 | Seq-R51 | W67299 | Greece | GRIN |
| *Pm21-C6* | MG831534 | Seq-R52 | 01C2300013* | Greece | GRIN-Czech |
| *Pm21-C7* | MG831535 | Seq-R53 | W67293 | Greece | GRIN |
| *Pm21-D1* | MG831557 | Seq-R54^k^ | GRA895 | Unknown | GBIS-IPK |
|  |  | Seq-R55^k^ | GRA1106 | Italy | GBIS-IPK |
|  |  | Seq-R56^k^ | GRA962** | Unknown | GBIS-IPK |
| *Pm21-D2* | MG831558 | Seq-R57 | PI 598396 | Greece | GRIN |
| *Pm21-D3* | MG831559 | Seq-R58 | PI 598395 | Greece | GRIN |
| *Pm21-D4* | MG831560 | Seq-R59 | PI 598398 | Greece | GRIN |
| *Pm21-E1* | MG831550 | Seq-R60 | W67270* | Greece | GRIN |
| *Pm21-E2* | MG831553 | Seq-R61 | PI 368886* | Turkey | GRIN |
| *Pm21-F1* | MG831549 | Seq-R62^l^ | GRA2717 | Greece | GBIS-IPK |
|  |  | Seq-R63^l^ | GRA2711* | Greece | GBIS-IPK |
|  |  | Seq-R64^l^ | GRA2714 | Greece | GBIS-IPK |
|  |  | Seq-R65^l^ | GRA2716* | Greece | GBIS-IPK |
| *Pm21-F2* | MG831551 | Seq-R66^m^ | PI 636504 | Greece | GRIN |
|  |  | Seq-R67^m^ | W67290 | Greece | GRIN |
| *Pm21-F3* | MG831552 | Seq-R68 | PI 251478* | Turkey | GRIN |
| *Pm21-F4* | MG831554 | Seq-R69 | GRA2716* | Greece | GBIS-IPK |
| *Pm21-G1* | MG831555 | Seq-R70^n^ | GRA1113 | Italy | GBIS-IPK |
|  |  | Seq-R71^n^ | GRA2736 | Greece | GBIS-IPK |
|  |  | Seq-R72^n^ | GRA1109* | Italy | GBIS-IPK |
| *Pm21-G2* | MG831556 | Seq-R73 | GRA1114* | Italy | GBIS-IPK |
| *Pm21-NF1* | MH184801 | Seq-S1 | GRA2738** | Greece | GBIS-IPK |
| *Pm21-NF2* | MH184802 | Seq-S2^o^ | GRA962** | Unknown | GBIS-IPK |
|  | MH184803 | Seq-S3^o^ | GRA1105** | Italy | GBIS-IPK |
|  | MH184806 | Seq-S4^o^ | DA6V#3 | Unknown | KSU |
| *Pm21-NF3* | MH184804 | Seq-S5^p^ | PI 598390** | Former Soviet Union | GRIN |
|  | MH184805 | Seq-S6^p^ | DA6V#1 | Unknown | GRIN |

^a~p^ The sequences marked with the same letter were identical.

^*^ The accessions that contained different copies of *Pm21* alleles.

^**^ The accessions, in which, individuals susceptible to powdery mildew were observed.

CBG, Cambridge Botanic Garden. GRIN, Germplasm Resources Information Network. GRIN-Czech, Germplasm Resources Information Network, Czech. GBIS-IPK, Genebank Information System of the IPK Gatersleben. NordGen, Nordic Genetic Resource Center. KSU, Kansas State University.
