# Supplementary material for: Genetic Diversity and Evolutionary Analyses Reveal the Powdery Mildew Resistance Gene Pm21 Undergoing Diversifying Selection
Source: Front Genet. 2020 May 12;11:489. doi: 10.3389/fgene.2020.00489 (PMC7241504; doi:10.3389/fgene.2020.00489)
Supplement: Table S2 — InDel polymorphisms in the Pm21 alleles. All InDels, compared with Pm21, occur after the positions showed in the brackets. [file Table_2.DOCX]

**Table S2.** InDel polymorphisms in the *Pm21* alleles. All InDels, compared with *Pm21*, occur after the positions showed in the brackets.

| InDel | DNA change (position) | Amino acid change (position) | Domain |
| --- | --- | --- | --- |
| In-1 | Insert TCCCCGCAAAA  AA(C)T(A)AACTCG(A)CG(C)CTCACGAG (753) | Insert SPQKI(Q)T  R(H)A(P)HE (251) | NB-ARC |
| In-2 | Insert GAG (1140) | Insert E (380) | NB-ARC |
| In-3 | Insert GTC (1442) | Change D to ES (481) | NB-ARC |
| In-4 | Insert CAG (1876) | Insert A (626) | LRR |
| Del-1 | Delete TAT (1023) | Delete Y (341) | NB-ARC |
| Del-2 | Delete AAT (1837) | Change EF to V (611) | LRR |
| Del-3 | Delete ACA (2074) | Change YT to S (691) | LRR |
