# Supplementary material for: Genetic Diversity and Evolutionary Analyses Reveal the Powdery Mildew Resistance Gene Pm21 Undergoing Diversifying Selection
Source: Front Genet. 2020 May 12;11:489. doi: 10.3389/fgene.2020.00489 (PMC7241504; doi:10.3389/fgene.2020.00489)
Supplement: Table S3 — Amino acid sites under positive selection. [file Table_3.DOCX]

**Table S3.** Amino acid sites under positive selection

| **Site** | **dN** | **dS** | **dN-dS** | **P-value** | **Model** |
| --- | --- | --- | --- | --- | --- |
| 628 | 4.53435 | 0 | 4.53435 | 0.03335 | F81 |
|  | 4.80998 | 0 | 4.80998 | 0.02657 | HKY85 |
|  | 4.69092 | 0 | 4.69092 | 0.02826 | TN93 |
|  | 4.69092 | 0 | 4.69092 | 0.02826 | GTR |
| 885 | 4.00174 | 0 | 4.00174 | 0.03297 | F81 |
|  | 4.48798 | 0 | 4.48798 | 0.02739 | HKY85 |
|  | 4.22173 | 0 | 4.22173 | 0.03417 | TN93 |
|  | 4.22173 | 0 | 4.22173 | 0.03417 | GTR |
| 903 | 5.46308 | 0 | 5.46308 | 0.01328 | F81 |
|  | 5.47458 | 0 | 5.47458 | 0.01272 | HKY85 |
|  | 5.47491 | 0 | 5.47491 | 0.01256 | TN93 |
|  | 5.47491 | 0 | 5.47491 | 0.01256 | GTR |
| 905 | 5.21377 | 0 | 5.21377 | 0.03727 | F81 |
|  | 5.72754 | 0 | 5.72754 | 0.00888 | HKY85 |
|  | 5.93430 | 0 | 5.93430 | 0.00569 | TN93 |
|  | 5.93430 | 0 | 5.93430 | 0.00569 | GTR |

F81, Felsensten 1981 model. HKY85, Hasegawa-kishino-Yano model. TN93, Tamura-Nei model. GTR: General Time Reversible model.
